# 

**Published:** 1891-09-12

**Authors:** 


[Registered at the General Post Office as a Newspaper for transmission in the United Kingdom and Abroad.]

WITH EXTRA NURSING SUPPLEMENT.

Per Annum, 6s. 6d.; post free.

Monthly Parts, 6d.; post free 8d.

Ditto per Annum, 8s., post free.

### THE HOSPITAL

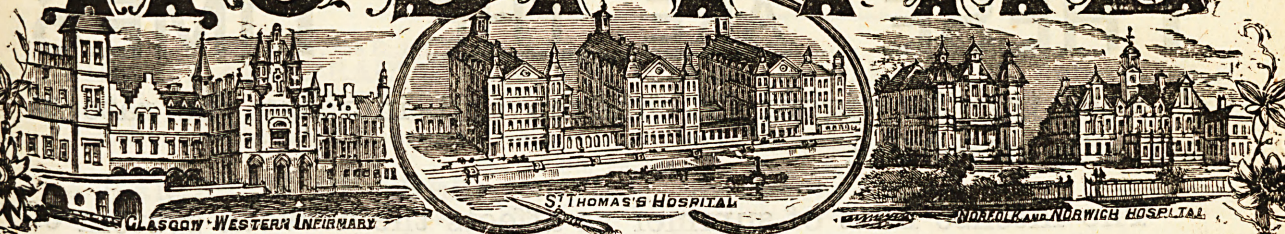

A WEEKLY JOURNAL OF

### Science, Medicine, Nursing and Philanthropy.

### SPECIAL STUDENTS' NUMBER.

### SATURDAY, SEPTEMBER 12th, 1891.

**The Histology of the Conscience** - - - - - - - 277

**The Doctor's Armchair.**—Medicine as a Calling - - 278

**How Doctors are Made** - - - - - - - - - 279

**The Practitioner's Mirror and Retrospect**—

MEDICINE.—On some Points in the Treatment of Diabetes— 283

II. SURGERY.—Diseases of the Fingers (*continued*). F—Abnormalities of Circulation - - - - - - - - 284

**Scotch Medical Schools.**—By a Scot - - - - - 281

**Annotations.**—The Workman and the Hospital—Lecturers, Note-takers, and Books - - - - - - - 282

**The Student of Medicine.**—Medical Schools and Degree-Granting Corporations—Syphilis: Its Transmission and Treatment (*continued*), 285; Appointments—Vacancies—Pass Lists - - - - - - - - - - 286

### EXTRA SUPPLEMENT—THE NURSING MIRROR.

**En Passant.**—The Pension Fund—Glasgow Queen's Nurses—Modern Midwifery—Short Items—Stockton Nursing Association—The Nurse and the Undertaker - - cxxxvii

**Lectures on Surgical Ward Work and Nursing.** By ALEX. MILES, M.D. Edin., F.R.C.S.E. Lecture XXXV.—Knives - - - - - - - - - - - cxxxviii

**The Princess of Wales and the Nurses** - - cxxxviii

**The Island of Gigha** - - - - - - - - cxxxix

**Appointments** - - - - - - - - - cxxxix

**For Reading to the Sick**—Plain Sailing - - - cxxxix

**Notes from Australia** - - - - - - - - cxl

**Presentation** - - - - - - - - - - cxli

**Wants and Workers** - - - - - - - - - cxli

**Everybody's Opinion.**—Water Beds—Three Months' Certificates—Poor Spinsters - - - - - - - - cxli

**Notes and Queries** - - - - - - - - - cxli

**Amusements and Relaxation** - - - - - - cxli

**Off Duty.**—Thrown Away - - - - - - - cxlii

